# Weak and Strong Gels and the Emergence of the Amorphous Solid State

**DOI:** 10.3390/gels4010019

**Published:** 2018-02-23

**Authors:** Jack F. Douglas

**Affiliations:** Materials Science and Engineering Division, National Institute of Standards and Technology, Gaithersburg, MD 20899, USA; jack.douglas@nist.gov; Tel.: +1-301-975-6779

**Keywords:** weak gel, strong gel, jelly, amorphous solidification, fractional differential equations, softness, shear thinning, aging, stretched exponential relaxation, Andrade creep

## Abstract

Gels are amorphous solids whose macroscopic viscoelastic response derives from constraints in the material that serve to localize the constituent molecules or particles about their average positions in space. These constraints may either be local in nature, as in chemical cross-linking and direct physical associations, or non-local, as in case of topological “entanglement” interactions between highly extended fiber or sheet structures in the fluid. Either of these interactions, or both combined, can lead to “gelation” or “amorphous solidification”. While gels are often considered to be inherently non-equilibrium materials, and correspondingly termed “soft glassy matter”, this is not generally the case. For example, the formation of vulcanized rubbers by cross-linking macromolecules can be exactly described as a second order phase transition from an equilibrium fluid to an equilibrium solid state, and amorphous solidification also arises in diverse physical gels in which molecular and particle localization occurs predominantly through transient molecular associations, or even topological interactions. As equilibrium, or near equilibrium systems, such gels can be expected to exhibit universal linear and non-linear viscoelastic properties, especially near the “critical” conditions at which the gel state first emerges. In particular, a power-law viscoelastic response is frequently observed in gel materials near their “gelation” or “amorphous solidification” transition. Another basic property of physical gels of both theoretical and practical interest is their response to large stresses at constant shear rate or under a fixed macroscopic strain. In particular, these materials are often quite sensitive to applied stresses that can cause the self-assembled structure to progressively break down under flow or deformation. This disintegration of gel structure can lead to “yield” of the gel material, i.e., a fluidization transition, followed by shear thinning of the resulting heterogeneous “jelly-like” fluid. When the stress is removed, however, the material can relax back to its former equilibrium gel state, i.e., gel rejuvenation. In contrast, a non-equilibrium material will simply change its form and properties in a way that depends on processing history. Physical gels are thus unique self-healing materials in which the existence of equilibrium ensures their eventual recovery. The existence of equilibrium also has implications for the nature of both the linear and non-linear rheological response of gel materials, and the present paper explores this phenomenon based on simple scaling arguments of the kind frequently used in describing phase transitions and the properties of polymer solutions.

## 1. Introduction

Gels are ubiquitous—many foods (e.g., gelatin, cheese, ketchup), consumer products (e.g., toothpaste, cosmetics, shaving cream, etc.) and industrial products (e.g., adhesives, asphalt) can be defined as belonging to a rheologically-defined class of materials termed “gels” (see gel reviews cited below). Moreover, the gel state is also characteristic of biological materials since the cytoplasm of eukaryotic cells is typically a gel [1], the cell as a whole has gel-like rheological properties [2], collagen extracts forms gels similar to those found in the cell extracellular matrix [3], and, in some cases, gel-like rheology extends to animal tissues composed of ensembles of cells “glued” together by the extracellular matrix [4], and it is thus not surprising that diverse forms of biological material such as foods, biofilms, soils, etc. have a gel-like nature. 

Gels are then the quintessential form of soft matter, and there have correspondingly been numerous reviews of gels from different perspectives. For example, Ragovina et al. have recently reviewed the defining characteristics of polymer gels [5], and Raghavan and Douglas [6] have discussed the definition of gels from a more general standpoint, and they also discuss many specific examples. The present work mainly focuses on the “critical” regime in which a liquid transform at equilibrium into a solid material, regardless of the specific interactions giving rise to the particle or molecular localization underlying gelation. This dynamical transition has many special physical and mathematical features that are not generally appreciated by physical scientists working with gels, and the present paper focuses on summarizing results that are dispersed in the fundamental physics, mathematics, and experimental polymer science and colloidal science literatures. This discussion provides a general framework for defining gels and understanding some of their often universal properties. 

Gels are amorphous solids rather than fluids having a finite shear viscosity, despite their disorderly molecular organization and their capacity to exhibit viscoelastic stress relaxation. Many gels are composed of polymer networks of chemically cross-linked molecules, as in rubbers, but others involve extended polymeric structures having only physical associations [7]. Chemical and associative cross-links are not necessary, however, and some gels involve topological interactions between the particles or molecules that cause them to be localized by surrounding particles. The molecules or particles of such topological gels are normally highly extended in shape, but are also sufficiently complex in geometrical form or their intermolecular interactions to preempt the formation of liquid crystalline phases [6]. This last form of gel might also be termed a “nematic gel”, a type of gel discussed below. In each of these different types of gels, local constraints on the molecules or particles or interactions serve to localize the particles, leading to the emergence of a collective elastic response of the material on macroscopic dimensions so that the material can sustain a perturbing stress on long timescales, as in the case of crystals whose molecules alternatively adopt translationally ordered configurations. 

Gels have often been classified generally as “soft glassy matter” based on a presumption that they are inherently non-equilibrium materials, as often supposed for glass materials [8,9], but this is not generally true. For example, the formation of vulcanized rubbers by cross-linking macromolecules can be described as a true equilibrium phase transition having features in common to the emergence of superconductivity, superfluidity, other forms of matter exhibiting the emergence of a collective macroscopic response [10]. Even as equilibrium materials, gels exhibit universal viscoelastic properties under the thermodynamic conditions in which the gel state first emerges, and this dynamical phase transition generally gives rise to a power-law scaling of the shear stress relaxation function *G*(*t*) with time *t* (see below), along with other scaling properties associated with an underlying dynamic phase transition [10]. These scaling relations are often observed in real gel materials (general reviews on this phenomenon are indicated below), and lately these scaling relations have been ascribed generally to the non-equilibrium nature of gels [8,9]. The Soft Glassy Model of Sollich and coworkers [8,9] ascribes the commonly observed power-law relaxation in gel materials to an exponential distribution of barrier heights whose origin is unspecified. Although we recognize that many materials described as “gels” are in a non-equilibrium state, there is also evidence that many gels form by reversible association for which equilbrium thermodynamics provides an appropriate description and we restrict our attention in the present paper to this class of materials.

Another basic property of physical gels having both theoretical and practical interest, and which helps discriminate them from non-equilibrium gel materials, is their response to stress and other macroscopic perturbations. It is often the case that large stresses, e.g., shearing at a steady rate, will cause self-assembled molecular and particle structures to break down, but when the stress is removed the material will recover its former equilibrium state. In contrast, an inherently non-equilibrium material would simply evolve progressively with deformation, depending on how long and in the fashion in which the material is perturbed. Physical gels are thus “self-healing” materials, which practically means that the existence of equilibrium ensures the material will recover its “unperturbed” state, or some new equilibrium state, specified by the relevant thermodynamic conditions. On the other hand, this tansformation process can be extremely slow and non-equilibrium effects can be prevalent in the form of physical aging- the slow drift of the material properties over long timescales. 

Naturally, the general tendency of physical gels, and viscoelastic pre-gel solutions of self-assembled molecules and particles, to approach an equilibrium state after a sufficient time has many implications for both the linear and non-linear rheological properties of this class of materials, and the present paper explores this phenomenon based on scaling arguments of the kind frequently used in describing phase transitions and polymer solution properties. A previous paper [6] focused on the linear rheology characteristics of physical gels for which there are no physical or chemical cross-links, i.e., gels in which the emergence of a solid-like collective response arises from topological interactions between the particles or self-assembled structures. Here, we also focus on the effect shear flow on the properties of this type of physical gel. 

## 2. Definition of “Weak” and “Strong” Gels

Many materials have a highly compliant “jelly-like” consistency rather than the fully solid-like character of an elastic solid. This “squishy” rheological response, so useful in diverse applications and found in numerous natural materials, is evidently intermediate in some fashion between the ideal Newtonian fluid and Hookean elastic states. In these viscoelastic fluids, the elastic response is observed as a transient phenomenon so that the material relaxes its stress after long times- “long” often being a matter of human patience. Even crystalline solids will “flow” given enough time so that complete stress relaxation at long times in viscoelastic materials does not ensure the existence of a finite fluid viscosity. 

In the transition region between the fluid and solid states, a novel gel state that we term a “weak gels” arises in which the material has an infinite viscosity, while at the same time having a vanishing equilibrium shear modulus. An infinite viscosity only requires the stress to decay sufficiently slowly that the integral of the stress relaxation function *G*(*t*) diverges. Given the ubiquity of this peculiar “intermediate” form of matter, some discussion is helpful to appreciate its nature, and its rather ubiquitous occurrence.

The standard rheological characterization of the viscoelastic properties of complex fluids and gel materials is normally performed in the frequency domain where the material is subjected to a small oscillatory deformation and the stress response is measured. The most commonly reported properties are the “storage modulus” *G*′ and the “loss modulus” *G*′′, related to the shear relaxation function by Fourier transformation. A fully developed gel or “strong gel” has the property, *G*′ > *G*′′, where both moduli (especially *G*′) have the further property of being nearly independent of frequency *ω* over a large frequency range. As noted before, such an elastic response to shear deformation ultimately derives from the presence of localized particles or molecules that store the deformation energy of the stressed material over long timescales. The existence of a linear stress-strain relation (Hooke’s law) implies that the material has a finite equilibrium shear modulus, *G*.

The transition between a viscous fluid to a solid gel with a non-zero shear modulus, *G_o_* = *G*(*t*), is a progressive rheological transition, and many materials that we classify as being “gel-like” exist in an state in which both *G*′ and *G*′′ are strongly frequency dependent, although a well-defined shear modulus or viscosity cannot be defined or measured. The case of measuring the viscosity of self-assembled actin filament and microtubule solutions provides a good example of this non-linear rheological phenomenon [11]. Why is this behavior so common in “soft” materials?

As noted before, the formation of a solid at equilibrium involves the emergence of a large-scale collective response of the material to external perturbing forces that deform the material as a whole. This is a remarkable phenomenon, and has much in common mathematically to other emergent, and less taken for granted, collective phenomena such as superfluidity and superconductivity [10]. The mathematical nature of these emergent collective response is reflected in the functional form of the stress relaxation function near the thermodynamic conditions at which the collective gel state first emerges. In particular, the very existence of amorphous solidification at equilibrium, as in the familiar case of forming a rubber by cross-linking polymer molecules, requires that solidification occurs as a continuous dynamical phase transition [10]. Moreover, stress relaxation in systems undergoing amorphous solidification at equilibrium must occur as a power-law decay [5], reflecting the hierarchical clustering of the molecules or particle species of the material, and the corresponding emergence of power correlations in the strain field within the gel material. In simple mathematical terms, the onset condition for forming an equilibrium gel state requires the stress relaxation function *G*(*t*) to takes the form [10],

(1) $$G\left(t\right)\sim t^{-\mu },\;0<\mu <1,$$

so that the Fourier-transformed counterparts, *G*′ and *G*′′, are likewise power-laws in frequency, *ω*. The ratio, tan *δ* ≡ *G*′′/*G*′, quantifies the relative energy dissipation to storage contribution to the viscoelastic response, and this quantity is a constant [12,13,14,15] determined by *μ* (The exponent power *μ*, however, is not universal and takes different values for different types of gels). In heuristic terms, tan *δ* quantifies the “firmness” of the weak gel material [12,13,14,15,16]. McKinley and coworkers discuss many instructive examples of this type of weak gel [14,15,16].

Winter and coworkers [17,18] have appropriately emphasized the emergence of power-law scaling in *G*(*t*) as the defining condition for the emergence of the gel state. We next briefly consider the rather special mathematical nature of this type of this constitutive relationship. We term materials satisfying this constitutive relationship to a good approximation as being “weak gels”, and we next further explain the rationale for this terminology. 

As noted before, the shear viscosity of a material is simply the integral of *G*(*t*),

(2) $$\eta =\int_{0}^{\infty }G(\tau )\;d\tau ,$$

The integral of *G*(*t*) defined by Equation (1) is divergent integral so that the shear viscosity *η* of a weak gel is infinite. An infinite viscosity is a necessary condition for the solid state, and this property motivates calling materials exhibiting a power-law relaxation “gels”. On the other hand, we see that a power-law form of *G*(*t*) means that an applied stress will eventually decay to 0 at long times so that these materials “relax” as in the case of liquids, while at the same time their equilibrium shear modulus *G*(*t*→∞), or *G(ω =* 0), equals 0. “Weak gels” are evidently a form of matter that is ‘intermediate’ [12,13] between being a Newtonian liquid having a finite viscosity and a Hookean solid state having finite shear modulus. 

The intermediate physical nature of the “weak gel” state can quantified mathematically by inserting the power-law form of *G*(*t*) into the standard integral equation relating shear stress *σ* and strain rate $\dot{\gamma }$,

(3a) $$\sigma \left(t\right)=\left[\frac{S}{\Gamma \left(1-n\right)}\right]\;\int_{-\infty }^{t}k\left(t-\tau \right)\dot{\gamma }\;(\tau )d\tau ,\;k\left(t-\tau \right)=\left[|t-\tau {|}^{-n}/\Gamma (1-n)\right]$$

The “transport coefficient” in this constitutive equation, denoted by Winter and coworkers as *S* [17,18], evidently involves units that have a peculiar fractional power of time [12,13,14,15]. This is natural since this equation can be recognized in the mathematical literature as a (Riemann-Liouville) fractional differential equation [12,13,14,15,19,20,21],

(3b) $$\sigma \left(t\right)=\left[\frac{S}{\Gamma (1-n)}\right]\;D^{\mu }\;\dot{\gamma }\;\left(\tau \right)\;d\tau ,$$

where *μ* is the order (generally non-integer) of the differential operator D where *μ* ≡ 1 – *n*, and Γ denotes the gamma function. There is no mathematical derivation here, Equation (3b) is simply a consequence of the definition of the fractional order differential-integral operator, D (In Equation (3), the function on which the operator D acts is implicitly multiplied by a Heaviside step function *H*(*t*) for technical reasons [20,21]). This constitutive relation subsumes the constitutive relations of homogeneous liquid and solid condensed materials into a single unifying relationship that allows for the precise characterization of the intermediate forms of matter described above. In particular, the Hookean solid and Newtonian liquid, where the stress is proportional to the strain and rate of strain, respectively, correspond to the limits *μ* = −1 and *μ* = 0, respectively. There is then a precise mathematical sense in which the weak gel state represents an intermediate state of matter between Newtonian fluids and Hookean solids. Specifically, the exponent *μ* quantifies the degree of rheological “intermediacy” [12,13].

While weak gels are arguably only a transitionary condition on the way to a “proper” gel state in which the material acquired a non-zero equilibrium shear modulus *G*, this physical state, or something well approximating it, is a prototypical condition for numerous everyday forms of soft matter found in living systems, and in many materials encountered in manufacturing applications.

The high degree of “softness” of weak gels materials makes them susceptible to material change by perturbation and some of the most characteristic and useful properties of these materials derives from this sensitivity and their self-healing when perturbations are removed and the equilibrium state recovered. In the next section, we describe the nature of this highly non-linear response, and scaling arguments to describe the property changes in these highly “susceptible” disordered condensed materials.

## 3. Simple Models of the Non-Linear Rheology of Pre-Gels and Gels

Even “strong” physical gel materials having a finite shear modulus are often highly compliant materials that can interconverted into liquids upon application of steady shearing, or other perturbing field (electric, magnetic, pressure), that breaks down the self-assembled structures into its fundamental component particles or polymers. Upon “yield” or “material fluidization”, the gel “melts”, and the viscosity of the resulting fluid then becomes finite. Increasing the rate of shear further leads to progressive decrease of the effective shear viscosity as the material structure is progressively broken down under flow. Strong shear thinning, following yield, is ubiquitous in gel materials [5,22,23].

The capacity of physical gels to achieve a metastable equilibrium under steady- shear conditions can be expected to lead to many regularities in the flow properties when the gel forms through equilibrium self-assembly. These flow-induced, or deformation-induced properties, are often significantly different from the quiescent material. The structure of such materials often breaks down locally in space under flow, say near the wall of gel in a capillary that is induced to flow by introducing an applied pressure, which is certainly a complication in describing their material properties which are often dependent on time and spatial scales. At high rates of flow, this structural breakdown takes the form of shear-bands in which the gel near the capillary wall transforms *locally* into a fluidized state whose spatial extent depends on the rate of flow. In addition to shear banding, fracture may also occur instead of gel fluidization at rates of deformation that is sufficiently rapid that the gel can no longer sustain the buildup of stress as in the case of a linear elastic material. It is also possible for material homogeneity to be preserved under flow, at least in an average sense, so that that the imposition of flow to a gel or pre-gel material ultimately leads to a stationary state that can be treated as a quasi-equilibrium state. The present work restricts itself to a consideration of non-linear rheological effects that arise under this type of idealized condition. 

Shear thickening and structure formation can also occur in incipient gels (“jellies”) formed by self-assembling molecules or particles that form viscoelastic solutions. This phenomenon should be most prevalent under thermodynamic conditions under which a small change in the fluid flow rate can enhance the rate of molecular association more than rate of molecular dissociation [24,25]. Shear thickening of this kind has been particularly well documented in the case of worm-like micelle solutions, as reviewed by Walker [26], but this phenomenon has been often observed in protein solutions and other complex fluids composed of associating molecules. This important phenomenon is not discussed further below.

An evolution in the structure of pre-gel solutions and gels with time also arises if the temperature, concentration, or any other thermodynamically relevant variable, is suddenly changed [27]. This “aging” phenomenon leads to a corresponding change in the size of the self-assembled extended structures within the material in time, and the interactions between these self-assembled structures grows until the system achieves its new equilibrium state. Slow aging is characteristic of this class of materials, and the sensitivity of their structure to shear and other deformations often complicates their rheological characterization, even if a characterization in terms of thermodynamics is relevant to understanding their properties. We next consider some of the other non-linear rheological properties of these materials and some simple scaling arguments to rationalize these properties, based on the presumption of a uniform material state at equilibrium under steady-state deformation.

Before proceeding, it is emphasized that there is limited rigorous theory describing the non-linear rheological properties of viscoelastic fluids, and the scaling arguments given below should be considered only a simplified model of this complex many-body phenomenon. The treatment of the viscoelastic properties of fluids near their critical point for phase separation is an exception to this general situation, and below we test our scaling arguments in the case of this theoretically and experimentally well-studied class of complex fluids in which the non-linear viscoelasticity likewise derives from particle association and its modification under fluid flow. 

First, we consider the origin of the power-law shear thinning that is often observed in sheared gels formed by molecular or particle “entanglement”. The increase of the viscosity of the gel before it has “set” into a solid often arises from the formation of self-assembled structures having a polymeric or fiber structure within a dispersing solvent. A rather phenomenon arises in fluids near their critical point for phase separation where the shear breaks down dynamic clusters associated with incipient phase separation. Precise renormalization group (RG) calculations [28,29], and numerous experiments, are available that describe the shear-thinning and the viscoelasticity of these complex fluids. This is one of the few non-trivial model associating fluid system in which a rigorous theory of shear-thinning has been developed.). Since cluster breakdown under shear flow is common to both phase separation and supramolecular assembly in solution, we first apply our scaling arguments to critical fluids to see if we can recover the results of more elaborate RG calculations [28,29] by direct physical reasoning, combined with scaling arguments in terms of relevant hydrodynamic, thermodynamic, and hydrodynamic parameters describing phase separating fluids.

The shear viscosity *η* of fluids near their critical points for phase separation universally diverges upon approaching the critical point for phase separation with a power of the equilibrium correlation length, *ξ^x^* [28,29,30]. This is a general effect observed for micellar solutions, polymer solutions, simple mixtures such as methanol and cyclohexane, and well as in gases such as Xenon near their critical point [17]. The clusters alter the momentum diffusion in the fluid [31], i.e., the viscosity, in a way that directly scales with a correlation length quantifying the average cluster size. The fact that *η* does not depend on the direction of shearing in our rotational viscometer reminds us that the shear rate dependent viscosity and normal stresses are even functions of the shear rate, which we make dimensionless by multiplying by the relaxation time *τ* obtained from *G*(*t/τ*). We then focus on shear viscosity as a function shear rate, η[($\dot{\gamma }\tau$)^2^], which scales as *η_o_* ~ *ξ^x^* in the zero shear limit, $\dot{\gamma }\to 0$. The assumption that η($\dot{\gamma }$*τ*) is an analytic function of shear rate implies the existence a perturbative expansion in ($\dot{\gamma }\tau$)^2^,

(4) $$\eta \left(\dot{\gamma }\tau \right)=1+C\left[{\left(\dot{\gamma }\tau \right)}^{2}\right]+C{\left(\dot{\gamma }\tau \right)}^{2},$$

where *C* is an unspecified constant. We further assume in our scaling argument that shearing ultimately breaks up the clusters and makes the fluid homogeneous, which physically means that η($\dot{\gamma }$*τ*) must ultimately be independent of $\dot{\gamma }$ at high shear rates. This limit provides a physical condition to guide the resummation (“renormalization”) of the expansion for η($\dot{\gamma }$*τ*) to obtain [32],

(5) $$\eta \left(\dot{\gamma }\tau \right)=\eta_{o}\;{\left[1+A_{1}{\left(\dot{\gamma }\tau \right)}^{2}\right]}^{-\alpha },$$

where *C* = *αA*_1_ where *A*_1_ is an unspecified constant. (This functional form is well-known phenomenological Carreau equation [22], but where the exponent *α* is specified.) In a critical fluid context [28,29,30], the relaxation time τ, the lifetime of the dynamic critical clusters, scales as, $\tau \sim \xi^{z}$, where the relaxation time scaling exponent *z* equals, *z* = *d* + *x* or *z* (*d* = 3) = 3.065. Only one exponent *α* value in Equation (5) is consistent with the shear-rate independent limit of η($\dot{\gamma }$) for large $\dot{\gamma }$,

(6) α=x/2z=(x/2)/(3+x),

For a large reduced shear rate $\dot{\gamma }\tau$, η($\dot{\gamma }\tau$) then has the predicted power-law form,

(7) $$\eta \left(\dot{\gamma }\right)\sim {\left(\dot{\gamma }\right)}^{-x},$$

Remarkably, this scaling argument recovers the exact asymptotic power-law scaling relation derived from an infinite order renormalization group *ε*-expansion calculation by Onuki and Kawasaki [28,29]. Equation (5), and refinements of this relation [31], have proven to be quite useful in describing data in diverse critical fluids over the entire crossover range of shear rates where this expression embodies the physical effect of the breakdown of dynamic clusters under steady-shear flow [32].

It also seems reasonable to apply such “physical renormalization” arguments to physical gels, and complex pre-gel complex fluids, formed from polymeric and extended fiber or sheet structures in which the interactions leading to molecular and particle localization are topological rather than direct cohesive intermolecular interactions. The formation of lyotropic liquid crystals is a familiar phenomenon in suspensions of extended molecules and rod-like fibers or platelets from the work of Onsager [33], and many experimental and theoretical works following his pioneering work. It is perhaps not surprising, but not so well appreciated, that if sufficient disorder is introduced into fiber suspensions (e.g., the introduction of a sufficient concentration of added spheres [34] or links between pairs of rods having a random distribution, but fixed relative angles with respect to each other [35], then one obtains a kind of entropically-driven self-assembly phenomenon in which the rods form dynamic clusters as a stable thermodynamic state. Evidently, disorder pre-empts the liquid crystal phase transition, leaving behind a remnant rounded thermodynamic transition, as suggested also for glass-forming liquids. [36] Accordingly, we term this type of gel material a “nematic gel” when the topological interactions are sufficiently strong that a solid state emerges from the localized rod-like or platelet like structures within the material. From this perspective, the formation of physical gels from carbon nanotubes within polymer matrices [37], and from even exfoliated clays [38], is quite natural because of the “crumpled” nature of these nanoparticles, disorder normally pre-empting their liquid crystalline ordering. Of course, charge and multipole interactions can be an appreciable complication in the interactions that give rise to gel formation in suspensions of complex-shaped particles and polyelectrolytes.

In the case of melts of long flexible and semi-flexible polymers, the chain conformations likewise deviate appreciably from rods and molecular shape fluctuates between chains so it is quite understandable that these materials in their high temperature melt state do not form a nematic, or other liquid crystalline phases, because of the large conformational disorder of this class of molecules. However, topological interactions certainly exist between long polymers in the melt state, and it seems reasonable to consider whether dynamic chain clusters arise in association with the “entanglement” phenomenon found in these materials. Indeed, Douglas and Hubbard [32] introduced a model of polymer melt dynamics based on the view that “chain entanglement” is a kind of entropically-driven self-assembly. One of the important predictions of this theory is that the polymer chains should form dynamic clump-like chain clusters whose mass, and thus volume, should exhibit an exponential distribution so that the stress relaxation of the material should decay as a stretched exponential function with a stretching index, β = 3/5 [32]. These predictions accord well with subsequent direct experimental and simulation observation of rod-clusters clusters having an exponential mass distribution by Galanis et al. [34], and with corresponding experimental observations of stress relaxation having a stretching exponent near 3/5 in entangled polymer melts [39,40,41]. There is also direct evidence consistent with the formation of dynamic clusters at equilibrium in entangled flexible polymer solutions from dynamic light scattering [42], but in the melt state there is not sufficient density contrast to directly discern such structures. These proposed dynamic structures in “entangled” polymer fluids could be studied by simulation methods similar to those being used to identify dynamic particle clusters in glass-forming liquids [43], but such studies have not yet been performed so that the entanglement model of Douglas and Hubbard has not been tested computationally.

Stretched exponential relaxation is rather characteristic of relaxation in materials exhibiting supramolecular assembly in assembly in the “pre-gel” regime in which the assembled structures have not yet grown to the point where particle localization and gelation have fully occurred. This type of complex fluid relaxation is also observed in chemically cross-linked gels in the vicinity of the gelation transition [44]. The fluid in this pre-gel regime characteristically has a jelly-like consistency, and we may define a “jelly” as being be such an incipient gel. In physical gels, the stretched exponential relaxation is the natural consequence of the size polydispersity of the molecular and particle clusters and the condition of equilibrium which leads to a near-universal exponential mass distribution of cluster mass.

In addition to “clump-like” like entanglement clusters, the self-assembly of fiber-like and worm-like structures under relatively low concentration conditions, as observed, for example, in small molecule gelator molecules and worm-like micelles, leads to a stretching exponent close to 1/3 [45,46,47]. Notably, as these near one-dimensional clusters become large and persistent in form and the structural relaxation time correspondingly diverges, both stress relaxation and power law creep are well-approximated by a power-law having the particular index value of *β* = 1/3 [32]. Creep deformation under an applied stress having an index 1/3, which is termed “Andrade creep”, is observed in a wide range of condensed solid materials [48,49,50]. Douglas and Hubbard [32] have further argued that self-assembling systems that form polydisperse branched and sheet-like polymeric structures through self-assembly should exhibit β values near ½ so that *β* is predicted to depend on the dimension of the self-assembled structures in the complex pre-gel fluid. 

The entanglement model of Douglas and Hubbard [32] makes many other predictions such as the dependence of the critical molecular mass for entanglement with chain structure, the dependence of the shear viscosity and self-diffusion coefficient with on chain stiffness and polymer excluded volume interactions and space dimension, etc. Here we focus on the implications of this model for the shear thinking of the shear viscosity and normal stresses of flexible polymer melts based ion exactly the same type of reasoning as applied to critical fluids, with adaptation to the interactions causing the clustering being topological in nature. 

First, it is recalled that the dynamic cluster model of flexible polymer entanglement [32] predicts that the “terminal stress relaxation time” *τ* and fluid shear viscosity *η* ∝ *τ* of concentrated polymer solutions and polymer melts should scale with the polymer molecular mass *M*,

(8) $$\eta_{o}\left(“\mathrm{entangled}”\right)\sim \tau \;\sim M^{\frac{10}{3}},$$

in the limit of zero-shear rates and *d* spatial dimensions [32]. Shearing at high rates is presumed to lead to a break down of the presumed entanglement clusters, as in the case of critical clusters under shear flow, so that the chain contributions to relaxation should become independent of shear rate for large shear rates, the *M* scaling relation for the fluid shear viscosity *η* should reduce to the well-known scaling predictions of the Rouse model [51],

(9) η (Rouse)~M,

Assuming the Rouse theory provides an adequate description of entangled polymer melts of flexible polymers. [52] Repeating the arguments before for *η*($\dot{\gamma }$*τ*) leads to the crossover relation [53],

(10) $$\eta \left[{\left(\dot{\gamma }\tau \right)}^{2}\right]=\eta_{o}{\left\{1+A_{1}[{\left(\dot{\gamma }\tau \right)}^{2}]\right\}}^{-\alpha },\;\alpha =7/20$$

More generally, *α* is predicted by the nematic glass model [32] to depends on the fractal dimension of the chain *d_f_* (which is governed by chain swelling, topology and stiffness), along with spatial dimensionality *d*,

(11) $$\alpha =1-2d/\left(d_{f}+2\right)\;\left(d+2\right)]/2,$$

Note that this relation implies a smaller shear thinning exponent 3/10 for entangled stiff polymers (*d_f_* ≈ 1) and that the mass scaling exponent of the terminal relaxation time *τ* is predicted to be appreciably larger than in the case of flexible polymers. The fact that “entanglement” ineractions are particularly large in stiff polymers, and other extended particle systems for which is knotting is highly improbable, suggests that topological coupling interactions rather than knotting underlies the “entanglement” interaction.

**Figure 1:**
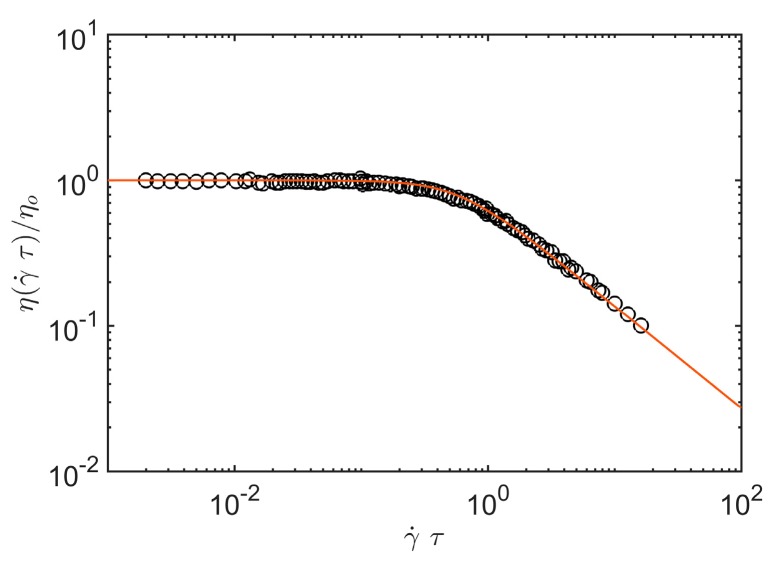
Shear viscosity *η*($\dot{\gamma }$*τ*) of concentrated “entangled” polystyrene solutions under steady-shear conditions as a function of reduced shear rate, $\dot{\gamma }$*τ*. Solid line denotes Equation (10) of the present paper where the data is taken from Graessely [54].

Figure 1 provides an illustrative comparison of shear viscosity data obtained by Graessley for sheared entangled concentrated polystyrene solutions where the chains can be taken to random coils (*d_f_* = 2) and *A*_1_ = 2.97 (See the review article of Graessely [54] for a pecification of the polymer mass, concentration and temperature range covered by these classical experiments.). 

The same arguments can be applied to the reduced compliance *J*($\dot{\gamma }$*τ*) of the polymer melt, the ratio of the first normal stress difference *N*_1_ to twice the square of the shear stress, *J*($\dot{\gamma }$*τ*) = *N*_1_/2 *σ*^2^, where σ is the shear stress. At low shear rates, this ratio equals the equilibrium shear compliance *J_o_* of an entangled material, which scales inversely to the plateau modulus, and at high rates the breakdown of the entangled chain clusters means that *J*($\dot{\gamma }$*τ*) must approach the limiting Rouse scaling, *J*($\dot{\gamma }$*τ*; Rouse) ~ *M*. For flexible polymers in three dimensions, the crossover scaling relation *J*($\dot{\gamma }$*τ*), consistent with these limits, is given by the crossover equation,

(12) $$J\left(\dot{\gamma }\tau \right)=J_{o}{\left\{1+A_{2}[{\left(\dot{\gamma }\tau \right)}^{2}]\right\}}^{\frac{3}{10}},$$

where *A*_2_ is a material specific constant.

**Figure 2:**
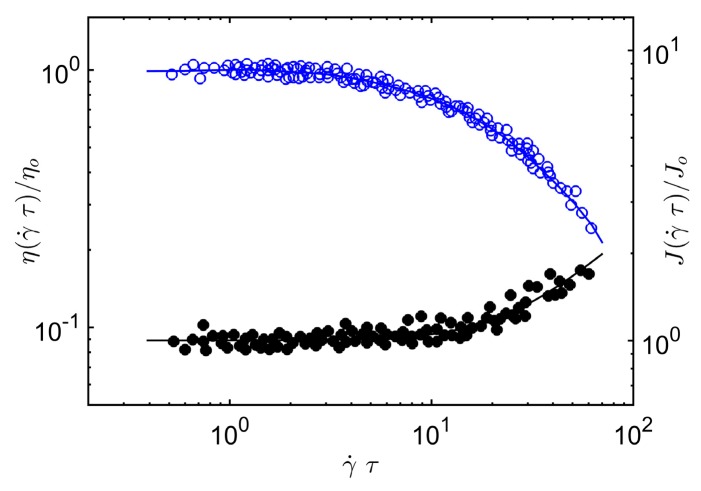
Shear viscosity *η*($\dot{\gamma }$*τ*) and compliance *J* ($\dot{\gamma }$*τ*) of “entangled” poly(alpha-methylstyrene) melt under steady-shear conditions as a function of reduced shear rate, $\dot{\gamma }$*τ*. Solid lines denote Equations (10) and (12) of the present paper and the data is taken from Sakai et al. [55].

In Figure 2, we compare Equation (12) and Equation (5) to *J*($\dot{\gamma }$*τ*) and *η*($\dot{\gamma }$*τ*) data on melts of entangled poly(α-methylstyrene). Again, we find that our scaling argument accords rather well with our scaling argument for the steady-shear shear viscosity and first normal stress of entangled concentrated poly(α-methylstyrene) solutions. See Sakai et al. [55] for a specification of the polymer mass, concentration and temperature range covered by these experiments.

The strong shear rate dependence of sheared gel and complex fluids (pre-gel solutions) properties is not restricted to the shear viscosity and extends to other properties characterizing the viscoelastic nature of these material such as first and second stress differences, *N*_1_ and *N_2_*. A simple scaling argument extending the arguments above allows for an estimate of the change of these properties with shear. In particular, we may estimate the second normal stress *N*_2_ of entangled polymer melts by invoking a physical picture of the particle or molecular clusters as being like elastic regions embedded in a background simple fluid. A treatment of this hydrodynamic problem indicates that the normal stress ratio *N*_2_/*N*_1_ equals—1/7 [56]. This ratio accords qualitatively with many observations on flexible polymer melts where a value near 0.2 is often observed [57]. We can then use the argument to estimate *N*_1_ to then estimate *N*_2_ for this type of complex fluid.

From this brief discussion, we can account for many of the linear and non-linear viscoelastic properties of complex associating fluids near their gel transition from a simple model that accounts for the breakdown of self-assembled particle clusters under steady flow. We note that recent theoretical studies of certain branched polymer (stars, starburst, dendrimer and [58,59,60] and nanoparticles with grafted polymer layers [61] form “topological gels” in which these “soft” polymeric particles undergo dynamic cluster formation and there is indeed experimental evidence of clustering of soft particles in solution [62]. Measurements on entangled ring polymer melts has also revealed the existence of power- law stress relaxation, consistent with weak gel rheology [63]. Since flexible polymers can like viewed as soft deformable particles in a coarse-grained perspective, it seems to extend this topological glass concept to linear polymer chains as well. There is clearly a close similarity between the nematic gel model and recent topological models of polymer entanglement, the main difference in these physical models is that the nematic gel model emphasizes the importance of average molecular shape of the extent of the topological coupling between the chains.

Networks of self-assembled molecules or particles in the fully developed gel state exhibit a wide range of non-linear properties under steady strain conditions and upon varying the rate of deformation without causing the material to enter the liquid state. Specifically, the elasticity of cross-linked flexible polymer solids, i.e., rubbers, is often characterized at moderate deformations by strain softening and a positive *N*_1_ while stiff fiber networks often exhibit strain stiffening and negative normal stresses under deformation [64]. The elasticity of these different classes of flexible polymer and stiff fiber gels could not be more different from each other. In a previous work [64], we introduced a simple model of the elasticity of flexible and stiff fiber networks and applied this model to strong gel materials. We also considered changes in the thermodynamic stability of “strong” gel networks with steady deformation such as strain induced “melting” based on simplified models. Comparison of special cases of our model to experimental observations on permanent and physical gels under diverse conditions show rather good agreement, indicating that these simplified models offer a practical approach to describing the elasticity of both rubbery cross-linked materials and thermally reversible gels formed by supramolecular assembly. To avoid repetition, the reader is referred to this previous paper [64].

## 4. Conclusions

Gels may form through a diversity of intermolecular interactions ranging from the formation of molecular and particle networks from the direct chemical cross-linking to physical gels arising from dynamic molecular and particle associations or predominantly topological interactions that likewise serve to localize particles and molecules in space on a long timescale- the essential physical factor underlying the formation of all gels, and indeed all solid materials. It is emphasized that amorphous solidification at equilibrium necessitates a “universal” power-law stress relaxation response near the point where the gel state first emerges [10] and the occurrence of this dynamical transition is suggested to rationalize the rather general observation of power-law relaxation near the gel point in diverse forms of condensed matter. This interpretation is in contrast to the Soft Glassy Matter model [8,9], which claims that the power-law relaxation of gel materials is an inherently out-of-equilibrium property of disordered materials. While it is difficult to definitively prove either interpretation of power-law relaxation, it is at least clear that equilibrium gels exhibiting power-law relaxation at their gel point exist. Future work should be aimed at interpreting the particular values of the power-law exponent observed in various types of gels, beyond existing work on chemically cross-linked gels based on percolation theory. 

The inherent “softness” of many physical gels, and the corresponding relatively weakness of the interactions that localize the molecules within these materials, also makes them generally susceptible to perturbations that can cause them evolve in time, allowing for a “tuning” of material properties in a way that depends on thermodynamic conditions and the magnitude and type of the applied perturbation. After cessation of these perturbations, the material can fully recover if allowed to come equilibrium. Since high driving forces normally break up all self-assembled structures when the forces are sufficiently strong, we can employ scaling arguments to effectively estimate how the steady state properties of these self-assembled gel materials evolve under shear, and other perturbations that strongly disrupt gel structure. The existence of an underlying equilibrium is crucial for exhibiting these responses, which are found in diverse real gel materials. It is hoped that this scaling arguments will provide a useful framework for organizing observations on gels and complex fluids and a conceptual foundation for developing a rigorous theory for the rheological properties of these ubiquitous soft materials.
